# Biofeedback control of photosynthetic lighting using real‐time monitoring of leaf chlorophyll fluorescence

**DOI:** 10.1111/ppl.70073

**Published:** 2025-01-21

**Authors:** Suyun Nam, Marc W. van Iersel, Rhuanito Soranz Ferrarezi

**Affiliations:** ^1^ Department of Horticulture University of Georgia Athens GA USA

## Abstract

Optimizing photosynthetic lighting is essential for maximizing crop production and minimizing electricity costs in controlled environment agriculture (CEA). Traditional lighting methods often neglect the impact of environmental factors, crop type, and light acclimation on photosynthetic efficiency. To address this, a chlorophyll fluorescence‐based biofeedback system was developed to adjust light‐emitting diode (LED) intensity based on real‐time plant responses, rather than using a fixed photosynthetic photon flux density (PPFD). This study used the biofeedback system to maintain a range of target quantum yield of photosystem II (Φ_PSII_) and electron transport rate (ETR) values and to examine if the adjustment logic (Φ_PSII_ or ETR‐based) and crop type influence LED light intensity. The system was tested in a growth chamber with lettuce (*Lactuca sativa*) ‘Green Towers’ and cucumber (*Cucumis sativus*) ‘Diva’ to maintain six ETR levels (30, 50, 70, 90, 110, 130 μmol·m^−2^·s^−1^) and five Φ_PSII_ levels (0.65, 0.675, 0.7, 0.725, 0.75) during a 16‐hour photoperiod. The ETR‐based biofeedback quickly stabilized the target ETR within 30–45 minutes, whereas the Φ_PSII_‐based system needed more time. The system adjusted light intensities according to target values, acclimation status, and crop‐specific responses. For example, to maintain a target ETR of 130 μmol·m^−2^·s^−1^, the gradual increase in Φ_PSII_ over time due to light acclimation allowed the required PPFD to decrease by 35 μmol·m^−2^·s^−1^. Lettuce showed higher photosynthetic efficiency and lower heat dissipation than cucumber, leading to higher PPFD adjustments for lettuce. This biofeedback system effectively controls LED light, optimizing photosynthetic efficiency and potentially reducing lighting costs.

## 1 INTRODUCTION

In plant production, artificial light is primarily categorized as either photoperiodic or photosynthetic light. Photoperiodic light, characterized by low‐intensity illumination, regulates flowering by extending day length (Runkle, 2015). In contrast, photosynthetic lighting is crucial for promoting crop growth and enabling year‐round production, which includes sole‐source and supplemental lighting. Indoor production systems, such as vertical farms, typically use sole‐source light‐emitting diodes (LED) to ensure uniform growth and cultivation of high‐value crops (Zhang et al., 2020). In greenhouse production, supplemental lighting is widely used when sunlight is limited or highly variable to meet daily light integral (DLI) requirements (Albright et al., 2000). Although photosynthetic lighting offers many advantages, its heavy reliance on electricity in controlled environment agriculture (CEA) systems can lead to both financial challenges and environmental concerns. For indoor vertical farming, sole‐source lighting can account for approximately 80% of the electricity demand, while in greenhouses, lighting costs can represent up to 30% of total energy expenses (Avgoustaki & Xydis, 2021; Watson et al., 2018). Therefore, optimizing photosynthetic light intensity to maximize crop production while minimizing energy use is essential.

Sole‐source lighting is used as the primary lighting source in indoor production, providing a steady photosynthetic photon flux density (PPFD) with a prolonged photoperiod throughout the growing cycle (Voutsinos‐Frantzis et al., 2023). Timers and on–off regimes can easily control supplemental light intensities. These strategies turn on the supplemental lighting during specific times of the day or activate the LEDs at full power only when the ambient PPFD drops below a certain threshold (Pinho et al., 2013). However, implementing more dynamic and energy‐efficient supplemental lighting control strategies is essential in greenhouse production due to fluctuating sunlight levels that can change within seconds.

The supplemental light intensity can also be precisely adjusted using pulse‐width modulation (PWM) LED drivers. PWM turns LED lights on and off at a very high frequency, allowing the fraction of time the LEDs are on, which enables dimmability of the LED array (van Iersel et al., 2016b). When a photosynthetic active radiation (PAR) sensor measures instantaneous variations in ambient light at the plant's canopy level, the dimmable LED array can adjust light intensity to maintain a specific PPFD threshold when the ambient light level falls below the threshold (Tennessen et al., 1995). This strategy, also known as “adaptive lighting control”, is simple and low‐cost, and it could save up to 20% in electricity consumption compared to traditional on–off regimes without significantly affecting biomass production (Pinho et al., 2013). Since photosynthetic light use efficiency decreases as PPFD increases, the adaptive lighting provides just enough light to reach the threshold, ensuring efficient use of supplemental light and making the system energy‐efficient and cost‐effective (van Iersel & Gianino, 2017).

However, photosynthetic efficiency is not only influenced by ambient light intensities but also by various environmental factors such as temperature, relative humidity, and carbon dioxide (CO_2_) concentration (Ball et al., 1987; Campbell et al., 1988; Oberhuber & Edwards, 1993). For example, high temperatures can limit photosynthetic capacity by reducing Rubisco activity, inhibiting chlorophyll biosynthesis, inactivating photosystem II (PSII), disorganizing thylakoid structures, and increasing energy dissipation (Baker & Rosenqvist, 2004; Mathur et al., 2014). Low temperatures can induce photoinhibition and reduce photosynthetic efficiency, particularly when combined with high light intensity (Zhou et al., 2019). Additionally, crop type and light acclimation can significantly affect the photosynthetic light use efficiency. Different crop species and cultivars exhibit varying photosynthetic capacities even under the same environmental conditions due to differences in leaf anatomy and photosynthetic machinery. High‐light‐adapted plants often have a higher quantum yield of photosystem II (Φ_PSII_) than shade‐adapted plants, indicating that photosynthetic efficiency may vary over time depending on the acclimation status (Zhen & van Iersel, 2017).

Supplemental lighting is usually controlled by predetermined and fixed light recipes with maintained PPFD and photoperiod, regardless of physiological changes and crop‐specific traits. Several studies proposed that real‐time photosynthetic properties can be used to optimize LED light intensity (Durmus, 2020; Schapendonk et al., 2006; van Iersel, 2017; van Iersel et al., 2016b; Weaver & van Iersel, 2019). Feedback loops can progressively update and tailor lighting recipes over time by deploying a sensing device to detect plant stress and photosynthetic performance, alongside a lighting control module. Chlorophyll fluorescence is a valuable tool for estimating overall photosynthetic performance (Kalaji et al., 2016). It offers significant advantages over CO_2_ gas exchange measurements, as it is relatively easy, non‐invasive, and instantaneous, without time‐consuming calibrations and stabilizing procedures (Maxwell & Johnson, 2000). Continuous measurement of chlorophyll fluorescence has the potential not only for monitoring environmental stress but also for optimizing the light environment (Ahlman et al., 2017; Kim et al., 2006).

van Iersel et al. (2016b) developed an innovative LED light control strategy that adjusts LED light intensities based on chlorophyll fluorescence parameters, known as a chlorophyll fluorescence‐based biofeedback (biofeedback) system. The biofeedback system measures Φ_PSII_ and electron transport rate (ETR) every 15 minutes and adjusts LED light intensity based on target Φ_PSII_ or ETR values whenever the measured chlorophyll fluorescence parameters differ from the target values. Using the ETR‐based biofeedback logic, an overall rate of photosynthesis can be maintained, and Φ_PSII_‐based biofeedback logic provides lighting only when plants can use it efficiently (van Iersel, 2017). Therefore, the biofeedback system has the potential to guarantee steady growth or improve energy efficiency compared to traditional light control strategies.

In the previous studies, the technical feasibility of the biofeedback system was tested with lettuce (*Lactuca sativa*) plants in a closed growth chamber, successfully maintaining both target Φ_PSII_ and ETR within a single day. However, the Φ_PSII_‐based logic gradually decreased and eventually turned off the LED lighting, while the ETR‐based logic unnecessarily upregulated PPFD due to excessive photoinhibition from repetitive saturation pulses (van Iersel et al., 2016a). Relatively high target ETR and low target Φ_PSII_ levels have only been tested, resulting in PPFD above 250 μmol·m^−2^·s^−1^ (van Iersel et al., 2016b). Therefore, a wide and closely spaced range of target Φ_PSII_ and ETR need to be tested to determine if the biofeedback system can adjust PPFD as precisely and reliably as other traditional lighting strategies. Additionally, the initial system used prototyping equipment that was over‐dimensioned, and appropriate settings for the frequency, intensity, and duration of saturating light pulses should be established to ensure that PPFD adjustments are based solely on target values while minimizing photoinhibition from the measurements themselves. Moreover, Φ_PSII_ and ETR‐based biofeedback systems have not yet been directly compared under the same conditions, and crop‐specific responses to PPFD adjustments with maintained target values have not been investigated.

Our objectives were 1) to validate the performance of the biofeedback system by maintaining a wide range of target Φ_PSII_ and ETR and adjusting PPFD accordingly, 2) to investigate the crop‐specific response to photosynthetic efficiency and the adjustment of LED light intensity, and 3) to provide a baseline for further research on the biofeedback system and to apply it to greenhouse conditions and larger‐scale CEA systems.

## 2 MATERIALS AND METHODS

### 2.1 Plant materials and experimental setup

Lettuce (*Lactuca sativa*) ‘Green Towers’ and cucumber (*Cucumis sativus*) ‘Diva’ seeds (Johnny's Selected Seeds) were sown into 10‐cm square containers filled with a peat‐perlite soilless substrate (Fafard 1P; SunGro Horticulture). Plants were germinated in a walk‐in growth chamber equipped with white LED light bars (red 39%, green 40%, blue 18%, far‐red 3%; RAY series with Physiospec indoor spectrum; Fluence Bioengineering). A 250 μmol·m^−2^·s^−1^ PPFD was provided at the canopy level for 16 hours, resulting in a DLI of 14.4 mol·m^−2^·d^−1^. The plants were irrigated daily using an automated ebb‐and‐flow subirrigation system with a 15 N‐2.2P‐12.4 K water‐soluble fertilizer solution with a nitrogen concentration of 100 mg·L^−1^ (Jack's Professional® LX 15–5‐15 Cal‐Mag LX; JR Peters). The average temperature, vapor pressure deficit (VPD), and CO_2_ level in the walk‐in growth chamber were 23.5 ± 0.2°C, 0.9 ± 0.2 kPa, and 818.4 ± 21.2 μmol·mol^−1^ (mean ± standard deviation).

**FIGURE 1:**
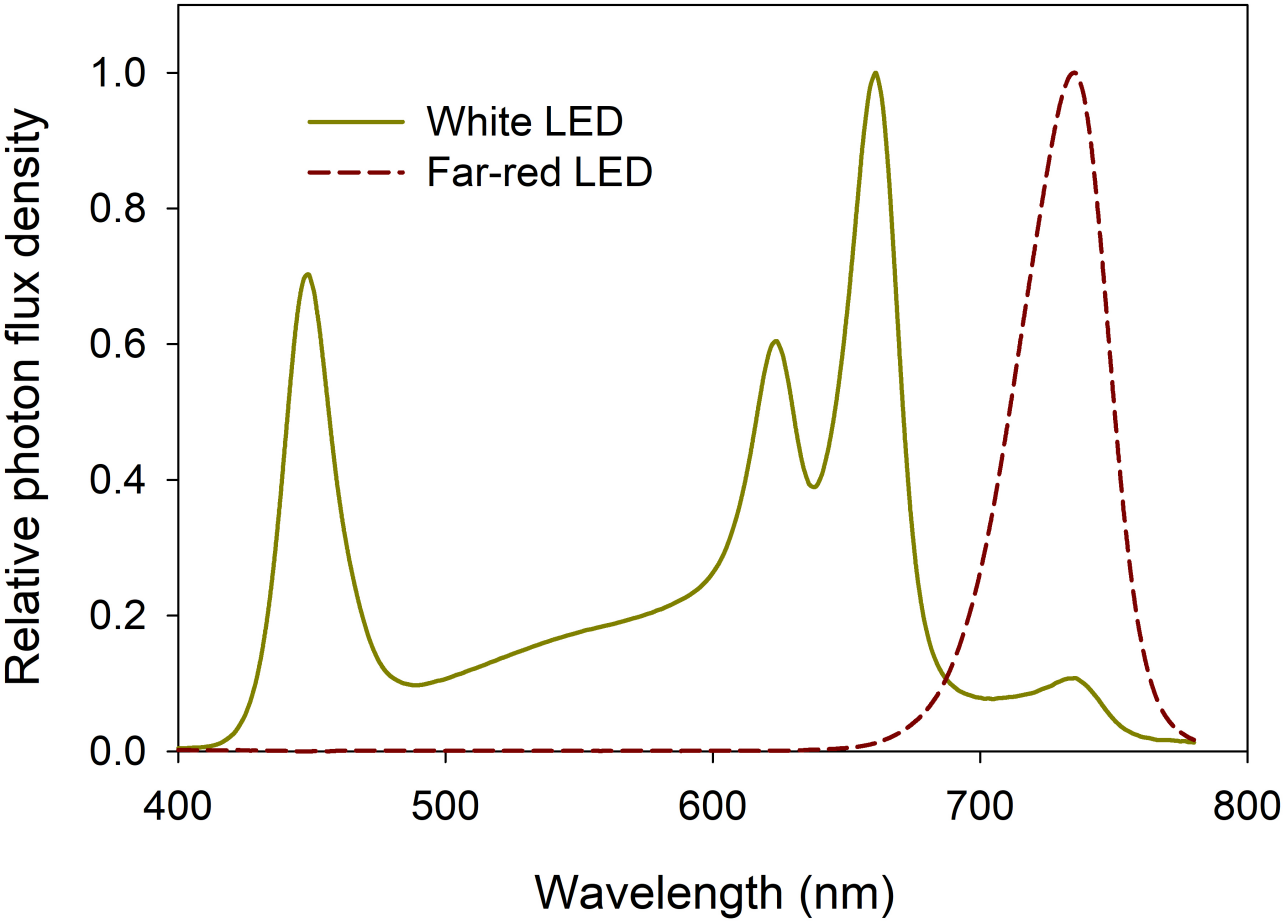
The spectral distribution of the white light‐emitting diode (LED) and the far‐red LED light measured 20 cm below the light fixtures using a spectroradiometer (LI‐180; LI‐COR Biosciences).

For the experiment, 4‐week‐old lettuce and 3‐week‐old cucumber plants were transferred from the walk‐in growth chamber to an experimental growth chamber (E15; Conviron), where the temperature was constantly maintained at 25°C. Ambient CO_2_ concentration was used, and the average VPD during the experiment was 1.7 ± 0.2 kPa. Before starting the experiment, each plant was hand‐irrigated with the same fertilizer solution. The growth chamber was divided into two experimental unit sections using a reflective aluminum wall to avoid disturbing the light environment. Six 200‐W white LED bars (red 50%, green 20%, blue 24%, far‐red 6%; Rev‐2; GrowRay) and one far‐red LED bar (738 ± 20 nm; Oslon SSL) were installed in each section. The spectral distributions of the white and far‐red LEDs were measured using a spectroradiometer (LI‐180; LI‐COR Biosciences) (Figure 1). The LED light modules were located 40 cm above the canopy, and a photoperiod of 16 hours (0:00–16:00) was used. Photodiodes (SLD‐69C1; Silonex) were used to measure PPFD in the growth chamber after calibration with a quantum sensor (MQ‐500; Apogee Instruments).

### 2.2 Chlorophyll fluorescence measurement

**FIGURE 2:**
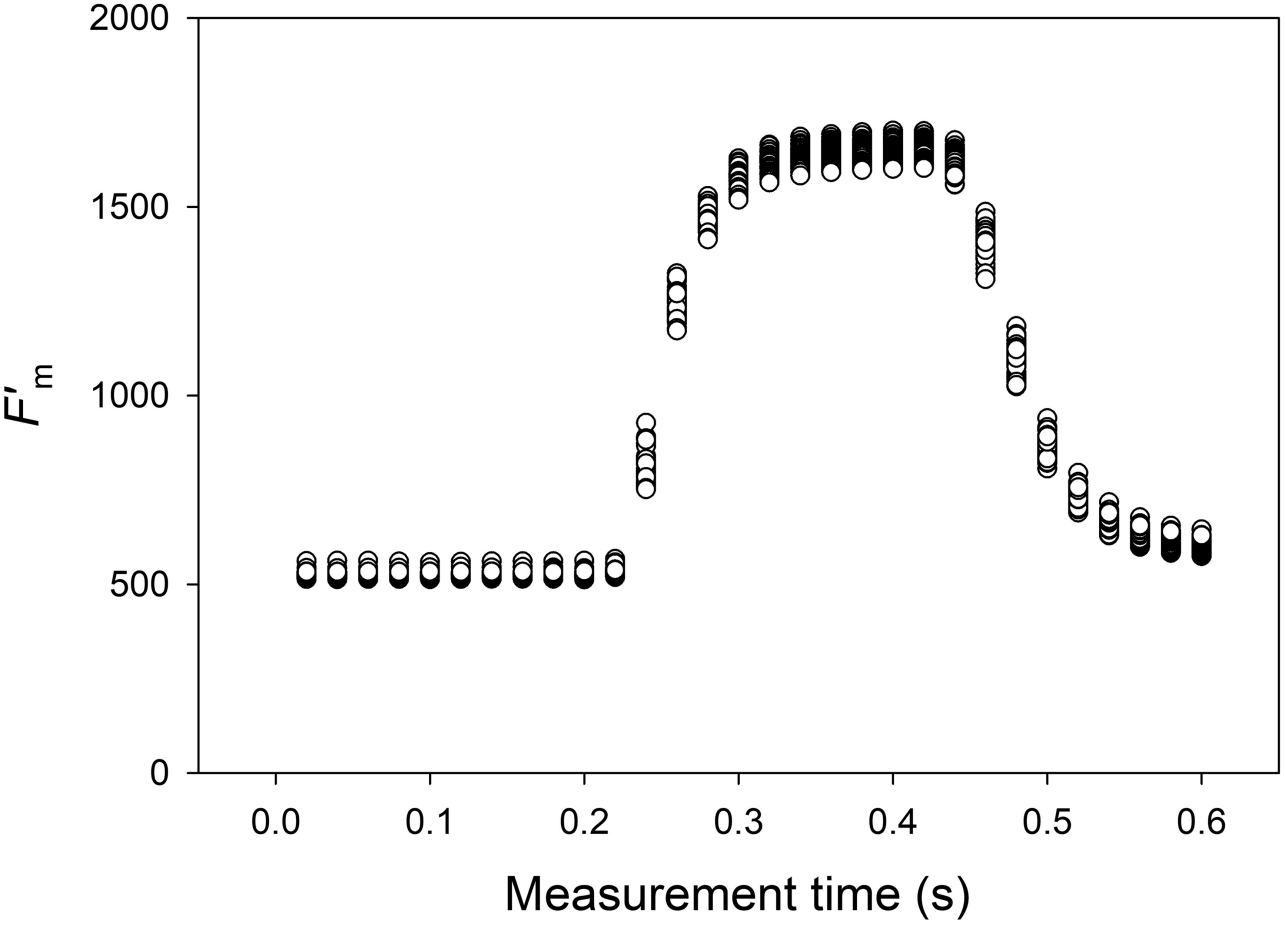
Changes in chlorophyll fluorescence yield recorded 30 times with 20 ms intervals during a saturating light pulse (*n* = 60). The highest value among them was determined as the maximum chlorophyll fluorescence (*F*'_m_) value, which is used to calculate the quantum yield of photosystem II (Φ_PSII_) and other chlorophyll fluorescence parameters.

The uppermost fully expanded leaves of lettuce and cucumber plants were selected to measure chlorophyll fluorescence using pulse‐amplitude modulated fluorometers (MINI‐PAM; Heinz Walz) in the growth chamber. During a 16‐hour photoperiod under the white LED light, saturating light pulses were applied every 15 minutes to measure maximum fluorescence in the light (*F'*_m_), and the steady‐state value of fluorescence (*F*_t_) was recorded immediately before the saturating light pulse. During the saturating light pulse, fluorescence yield was measured 30 times at 20‐ms intervals, and the highest value among them was recorded as *F'*_m_. The duration and intensity of the saturating light pulse were adjusted to the minimum necessary to ensure the fluorescence yield was fully saturated and reached a plateau (Figure 2).

The Φ_PSII_ was calculated as (*F'*_m_ – *F*_t_)/*F'*_m_ (Genty et al., 1989). Φ_PSII_ represents the proportion of the light energy absorbed by chlorophyll associated with PSII that is used for photochemistry. The linear ETR was estimated as ETR = Φ_PSII_ × PPFD × 0.5 × 0.84, assuming that photons are equally partitioned between PSI and PSII and that the leaf absorbs 84% of the incident PPFD (Maxwell & Johnson, 2000). Incident PPFD was measured by the photodiode at the canopy level, close to the leaf clip of the fluorometer. After the saturating light pulses, the white LED light was turned off for a couple of seconds, and the far‐red LED light was applied for a second. Darkening leaves with far‐red illumination allows the estimation of a zero level of fluorescence (*F'*_o_) in the light, which is essential for following calculations of chlorophyll fluorescence parameters (Maxwell & Johnson, 2000). Then, the far‐red LED light was turned off, and the white LED light was turned back on.

All plants were transferred from the walk‐in growth chamber to the experimental growth chamber at 20:00 and remained for 24 hours until 20:00 the next day. Dark‐adapted chlorophyll fluorescence (*F*_o_ and *F*_m_) was measured hourly before and after the light period. The maximum efficiency of PSII (i.e., the quantum efficiency if all PSII centers were open) was calculated as (*F*_m_ – *F*_o_)/*F*_m_ = *F*_v_/*F*_m_.

When the light energy is absorbed in PSII, it is utilized in three ways: 1) for photochemistry, contributing to the photosynthesis process, 2) dissipated as heat through non‐photochemical quenching (NPQ) mechanisms, or 3) dissipated through passive and non‐regulated processes. Φ_NPQ_ and Φ_NO_ represent the quantum yield of non‐photochemical quenching and the quantum yield of non‐regulated energy dissipation, respectively, where Φ_NPQ_ + Φ_NO_ + Φ_PSII_ = 1. Φ_NPQ_ was used instead of NPQ in this study because Φ_NPQ_ provides a normalized measure of NPQ and useful for comparing photosynthetic performance across different species or varying light intensities. Φ_NPQ_ and Φ_NO_ were calculated using the following equations:

$$\text{Photochemical quenching}\;\left(\mathrm{qP}\right)=\left({F^{'}}_{m}–F_{t}\right)/\left({F^{'}}_{m}–{F^{'}}_{o}\right)$$

$$\text{Coefficient of photochemical quenching}\;\left(qL\right)=qP\times \left({F^{'}}_{o}/F_{t}\right)$$

$$\mathrm{NPQ}=\left(F_{m}–{F^{'}}_{m}\right)/{F^{'}}_{m}$$

$$\Phi_{\mathrm{NO}}=1/\left(\mathrm{NPQ}+1+qL\times \left(\left(F_{m}/F_{o}\right)–1\right)\right)$$

$$\Phi_{\mathrm{NPQ}}=1–\Phi_{\text{PSII}}–\Phi_{\mathrm{NO}}$$

### 2.3 Biofeedback hardware and lighting control logic

The fluorometer was remotely controlled by a datalogger (CR1000; Campbell Scientific) via an RS‐232 interface. An RS‐232 interface cable connected the fluorometer's RS‐232 port to two communication (COM) ports on the datalogger. The fluorometer can be operated through a terminal program using custom software (GmbH, 1999). In this study, we utilized proprietary software (LoggerNet and CRBasic editor version 4.7; Campbell Scientific) for programming. Key communication parameters, such as baud rate, data bits, stop bits, and parity, were configured according to the fluorometer manual (GmbH, 1999).

Triggering measurements and data transfer were achieved by using specific sets of commands consisting of low‐case letters. For example, sending the command ‘m1’ activated the measuring light, while ‘s’ initiated a saturating light pulse. Commands for data transfer, such as ‘f’ for *F*_t_ and ‘fmp’ for *F'*_m_, allowed the datalogger to retrieve chlorophyll fluorescence values. Further calculations of the chlorophyll fluorescence parameters, such as ETR, Φ_PSII_, and *F*_v_/*F*_m_, were conducted by the datalogger using LoggerNet. Additional details on the RS‐232 communication protocol between the fluorometer and datalogger are available in GmbH (1999) and Yonghua (2012).

The datalogger was also connected to an analog output module (SDM‐A04; Campbell Scientific), which provided a 0–10 V DC signal to a control board that adjusted the PWM duty cycle of the dimmable LEDs. The duty cycle, ranging from 0 to 1, determined the proportion of time the LEDs were energized per cycle, thereby controlling light intensity. The biofeedback system dynamically adjusted the duty cycle to regulate PPFD in response to real‐time chlorophyll fluorescence measurements. When measured ETR or Φ_PSII_ deviated from target values, the biofeedback system recalculated the duty cycle every 15 minutes to bring the parameter back to the target. The adjustment followed these equations:

$$\mathrm{ETR}-\text{based}:\mathrm{New}\;\text{duty cycle}=\mathrm{Old}\;\text{duty cycle}+\left(\text{target}\;\mathrm{ETR}–\text{current}\;\mathrm{ETR}\right)/\left(\Phi_{\text{PSII}}\times 500\right)$$

$$\Phi_{\text{PSII}}-\text{based}:\mathrm{New}\;\text{duty cycle}=\mathrm{Old}\;\text{duty cycle}+\left(\text{current}\;\Phi_{\text{PSII}}–\text{target}\;\Phi_{\text{PSII}}\right)/10$$

For the ETR‐based equation, the difference between the target and current ETR was added to the previous duty cycle, enabling increases in PPFD for insufficient ETR and decreases for excessive ETR, as PPFD and ETR are positively correlated. In contrast, the Φ_PSII_‐based equation subtracted the difference between the target and current Φ_PSII_ from the previous duty cycle, as PPFD and Φ_PSII_ are negatively correlated (Weaver & van Iersel, 2019). Thus, a lower Φ_PSII_ compared to the target led to a reduction in PPFD. To ensure appropriate scaling within the 0–1 duty cycle range, these differences were divided by specific coefficients. Larger coefficients resulted in slower adjustments, while smaller coefficients could cause oscillations, preventing stabilization of target values. Through preliminary trials, the coefficients were carefully optimized to balance stability and responsiveness in duty cycle adjustments. The equations were embedded in the datalogger program for automated calculations, with updated duty cycle values transmitted to the control board via the analog output module.

### 2.4 Light response curve

In addition to the biofeedback trial, light response curves of lettuce and cucumber at the same growth stage were obtained to investigate crop‐specific photosynthetic efficiency in a short‐term period. All plants were dark‐adapted for 1 hour before measurements. Chlorophyll fluorescence parameters (ETR, Φ_PSII_, Φ_NPQ_, and Φ_NO_) were collected from the uppermost fully expanded leaves using the fluorometer across a wide range of PPFD values. The light response curves were measured in the same growth chamber, while the temperature was consistently maintained at 25°C. PPFD increased in 20 steps from 20 to 750 μmol·m^−2^·s^−1^ (20, 35, 50, 75, 100, 125, 150, 175, 200, 250, 300, 350, 400, 450, 500, 550, 600, 650, 700, and 750 μmol·m^−2^·s^−1^), with each PPFD step maintained for 15 minutes for acclimation. Chlorophyll fluorescence parameters were recorded at the end of each step. Measurements were conducted with four replicates for each crop. The maximum PPFD of 750 μmol·m^−2^·s^−1^ was achieved using the full power of the LED modules at the canopy level.

### 2.5 Experimental setup and statistical analysis

Six levels of target ETR (30, 50, 70, 90, 110, and 130 μmol·m^−2^·s^−1^) and five levels of target Φ_PSII_ (0.65, 0.675, 0.7, 0.725, and 0.75) were maintained for 16 hours for both lettuce and cucumber using the ETR‐based and Φ_PSII_‐based biofeedback systems. Each target treatment, with four replications, was randomized within one of two sections of the growth chamber, with a single plant per crop used for each experimental unit. Adjusted PPFD and chlorophyll fluorescence parameters (ETR, Φ_PSII_, and Φ_NPQ_) were recorded every 15 minutes to the datalogger. Statistical analysis was performed using a two‐way analysis of variance (ANOVA) to determine significant differences in chlorophyll fluorescence parameters among crop types and PPFD levels. Differences in *F*_v_/*F*_m_ among dark adaptation hours were tested with one‐way ANOVA. Tukey's Honestly Significant Difference (HSD) test was employed for mean separation in R software (version 4.4.1; R Foundation for Statistical Computing) at a 95% confidence level.

## 3 RESULTS

### 3.1 Performance of the biofeedback systems in maintaining target ETR and Φ_PSII_

**FIGURE 3:**
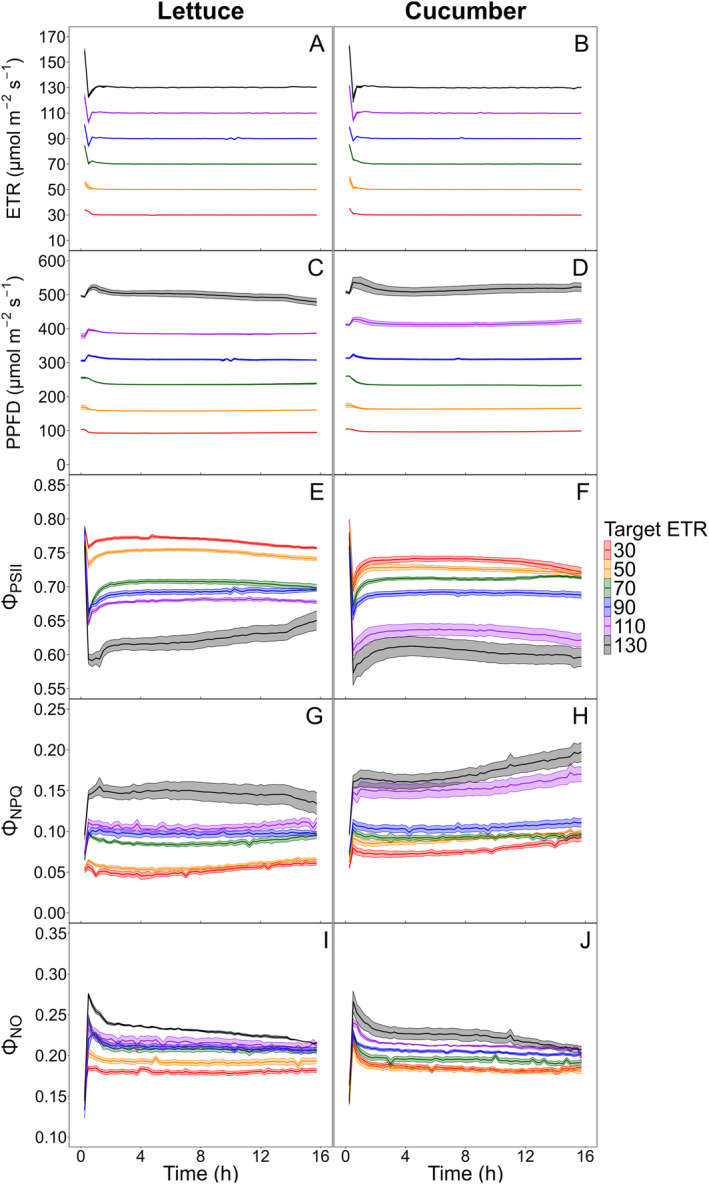
Six levels of target electron transport rate (ETR) (30, 50, 70, 90, 110, and 130 μmol·m^−2^·s^−1^) were maintained for a 16‐hour photoperiod using lettuce (*Lactuca sativa*) ‘Green Towers’ (A) and cucumber (*Cucumis sativus*) ‘Diva’ (B). A biofeedback system adjusted the photosynthetic photon flux density (PPFD) based on these target values (C and D). Data were collected for the quantum yield of photosystem II (Φ_PSII_) (E and F), quantum yield of non‐photochemical quenching (Φ_NPQ_) (G and H), and quantum yield of non‐regulated energy dissipation (Φ_NO_) (I and J). Solid line and shaded area indicate the mean and standard error (*n* = 4) of each target ETR treatment, respectively, recorded at 15‐minute intervals. Some standard error areas are not visible due to their minimal sizes.

ETR and Φ_PSII_ were measured every 15 minutes of each biofeedback cycle, and current ETR and Φ_PSII_ values were compared with the target values. LED light intensities were adjusted during every cycle based on the differences between current and target values. PPFD increased slightly when the current ETR was lower than the target, or the current Φ_PSII_ was higher than the target, and vice versa. For the ETR‐based biofeedback logic, only 2–3 biofeedback cycles (30–45 minutes) were needed for the current ETR to reach the target values (30, 50, 70, 90, 110, and 130 μmol·m^−2^·s^−1^), and these values were maintained consistently throughout the day (Figure 3A,B). Regardless of the target ETR level, all ETR values were stable, with minimal standard errors among the four replicates, demonstrating that the biofeedback system controls ETR with high accuracy and precision.

**FIGURE 4:**
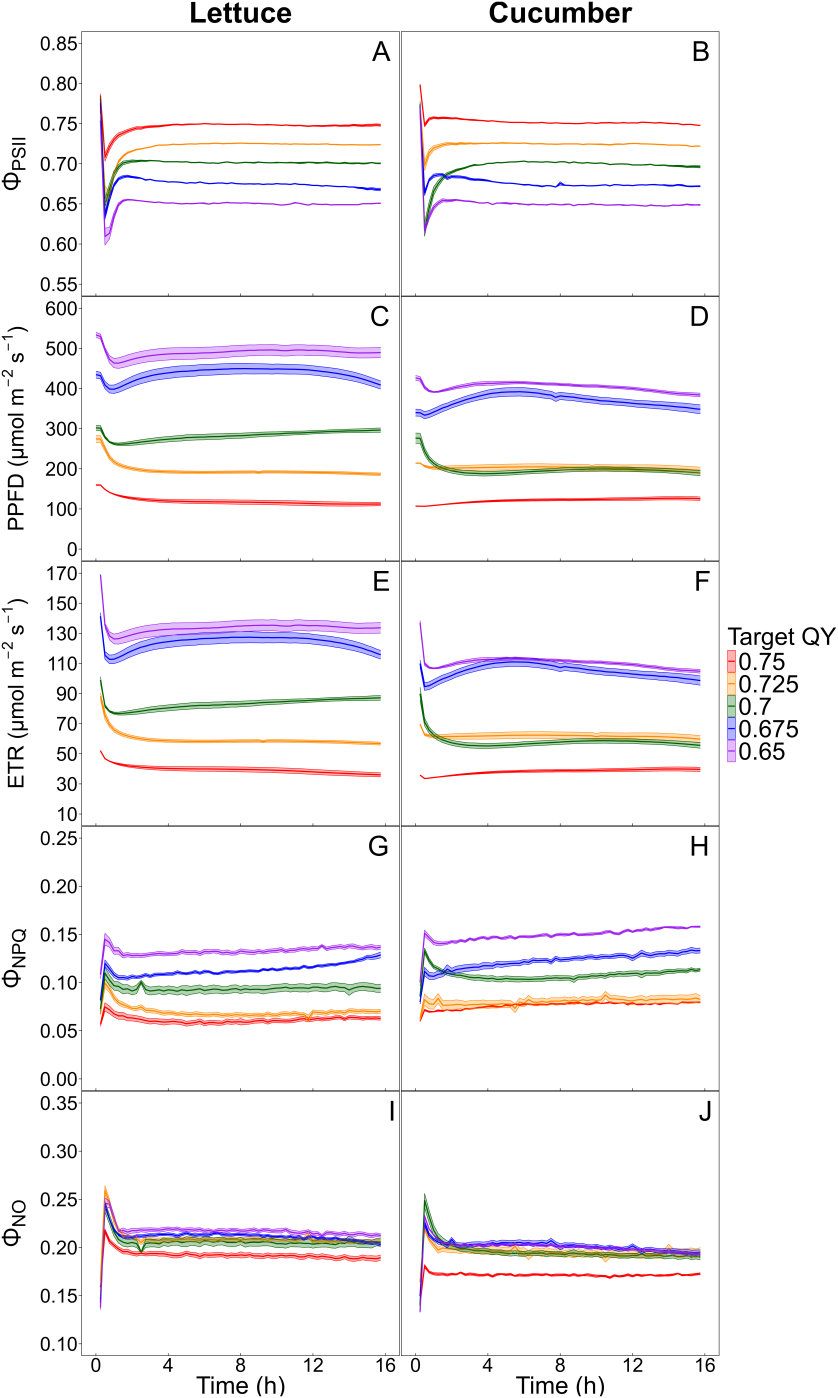
Five levels of target quantum yield of photosystem II (Φ_PSII_) (0.65, 0.675, 0.7, 0.725, and 0.75) were maintained for a 16‐hour photoperiod using lettuce (*Lactuca sativa*) ‘Green Towers’ (A) and cucumber (*Cucumis sativus*) ‘Diva’ (B). A biofeedback system adjusted the photosynthetic photon flux density (PPFD) based on these target values (C and D). Data were collected for the electron transport rate (ETR) (E and F), quantum yield of non‐photochemical quenching (Φ_NPQ_) (G and H), and quantum yield of non‐regulated energy dissipation (Φ_NO_) (I and J). Solid line and shaded area indicate mean and standard error (n = 4) of each target ETR treatment, respectively, recorded at 15‐minute intervals. Some standard error areas are not visible due to their minimal sizes.

The Φ_PSII_‐based biofeedback system also achieved and maintained the target Φ_PSII_ values (0.65, 0.675, 0.7, 0.725, and 0.75). However, the lighting control logic required 4–8 cycles (1–2 hours) to reach these target values (Figure 4A,B), approximately twice as long as the time needed for the ETR‐based logic (Figure 3A,B). Increasing the frequency of the biofeedback cycles in the initial hours can reduce the time required to achieve the target Φ_PSII_. As well as the ETR‐based logic, the Φ_PSII_‐based biofeedback system maintained stable Φ_PSII_ with minimal standard errors across replications.

### 3.2 Adjustment of the LED light intensities based on target values

When the LED light was first turned on, Φ_PSII_ remained relatively high (0.776 ± 0.013) during the initial first cycle, regardless of target treatments (Figures 3E,F and 4A,B). This high initial Φ_PSII_ may have led to excessive or fluctuating adjustments in LED light intensities during the early cycles. To mitigate this, predetermined PPFD values for each treatment were maintained for two cycles (30 minutes) to allow the plants to acclimate to the abrupt lighting changes from darkness. The predetermined PPFD values were set to 100, 150, 250, 300, 375, and 500 μmol·m^−2^·s^−1^ for target ETR levels of 30, 50, 70, 90, 110, and 130 μmol·m^−2^·s^−1^, respectively. Also, the predetermined PPFD values were set to 100, 250, 300, 400, and 500 μmol·m^−2^·s^−1^ for target Φ_PSII_ levels of 0.75, 0.725, 0.7, 0.675, and 0.65, respectively. During the experiment, environmental factors such as temperature, humidity, and CO_2_ concentration were kept constant to assess whether target chlorophyll fluorescence levels and light acclimation status influenced the adjustment of PPFD.

The target ETR levels, ranging from 30 to 130 μmol·m^−2^·s^−1^, adjusted the PPFD between 100 and 500 μmol·m^−2^·s^−1^. PPFD was inversely proportional to Φ_PSII_ while maintaining stable ETR, as ETR is a product of Φ_PSII_, PPFD, and fixed coefficients. For most ETR treatments, both PPFD and Φ_PSII_ achieved specific levels and remained stable throughout the day due to the consistent environmental conditions in the growth chamber (Figure 3). Each target treatment resulted in nearly evenly spaced adjusted PPFD ranges, reflecting the inherent relationship between PPFD and ETR in the equation. However, subtle changes in Φ_PSII_ over time led to minor adjustments in PPFD, especially under high ETR treatments. All target ETR levels for lettuce and cucumber initially increased Φ_PSII_ within a couple of hours, leading to a reduction in PPFD. Further, target ETR 130 for lettuce substantially increased Φ_PSII_ from 0.60 to 0.65 during the photoperiod, causing PPFD to decrease from 515 to 480 μmol·m^−2^·s^−1^. However, for cucumber, target ETR levels of 110 and 130 resulted in a slight decrease in Φ_PSII_ and a corresponding increase in PPFD over time.

In the ETR‐based logic, the PPFD ranges were evenly spaced and remained stable over time because ETR includes PPFD in its calculation. While the target ETR was kept constant, the PPFD adjusted stably and linearly according to the target ETR values. In contrast, the Φ_PSII_‐based biofeedback logic demonstrated more dynamic control of LED light intensities than the ETR‐based logic. Lower target Φ_PSII_ levels required higher PPFD, whereas higher target Φ_PSII_ levels had lower PPFD due to the increased efficiency of light energy utilization by plants at low light conditions. Target Φ_PSII_ levels from 0.75 to 0.65 adjusted PPFD in the ranges of 100–500 μmol·m^−2^·s^−1^ for lettuce and 100–400 μmol·m^−2^·s^−1^ for cucumber. Lettuce had relatively higher PPFD than cucumber under the same target Φ_PSII_ values between 0.65 and 0.7, indicating greater photosynthetic efficiency in lettuce (Figure 4). The adjusted PPFD ranges were not evenly spaced with respect to target Φ_PSII_ values, and for cucumber, PPFD levels were mostly overlapped at target Φ_PSII_ values of 0.7 and 0.725. Despite the biofeedback logic maintaining consistent Φ_PSII_, the adjusted PPFD for some treatments (Φ_PSII_ 0.675 and 0.7 for lettuce; 0.675 for cucumber) varied over time, suggesting that photosynthetic efficiency was influenced by both target Φ_PSII_ levels and temporal changes.

### 3.3 Crop‐specific differences in photochemical efficiency and heat dissipation

Since Φ_PSII_, Φ_NPQ_, and Φ_NO_ compete for absorbed light energy, variations in Φ_PSII_ are attributed to the changes in two types of heat dissipation mechanisms, Φ_NPQ_ and Φ_NO_. For lettuce, a target ETR of 130 μmol·m^−2^·s^−1^ with high PPFD gradually increased Φ_PSII_ over time, while both Φ_NPQ_ and Φ_NO_ decreased. Conversely, for cucumber, a target ETR of 110 and 130 μmol·m^−2^·s^−1^ led to a decrease in Φ_NO_ but a more significant increase in Φ_NPQ_, resulting in a slight reduction in Φ_PSII_ (Figure 3). Since the Φ_PSII_‐based biofeedback logic kept Φ_PSII_ levels constant, Φ_NPQ_ and Φ_NO_ levels were also maintained stable for both lettuce and cucumber.

**FIGURE 5:**
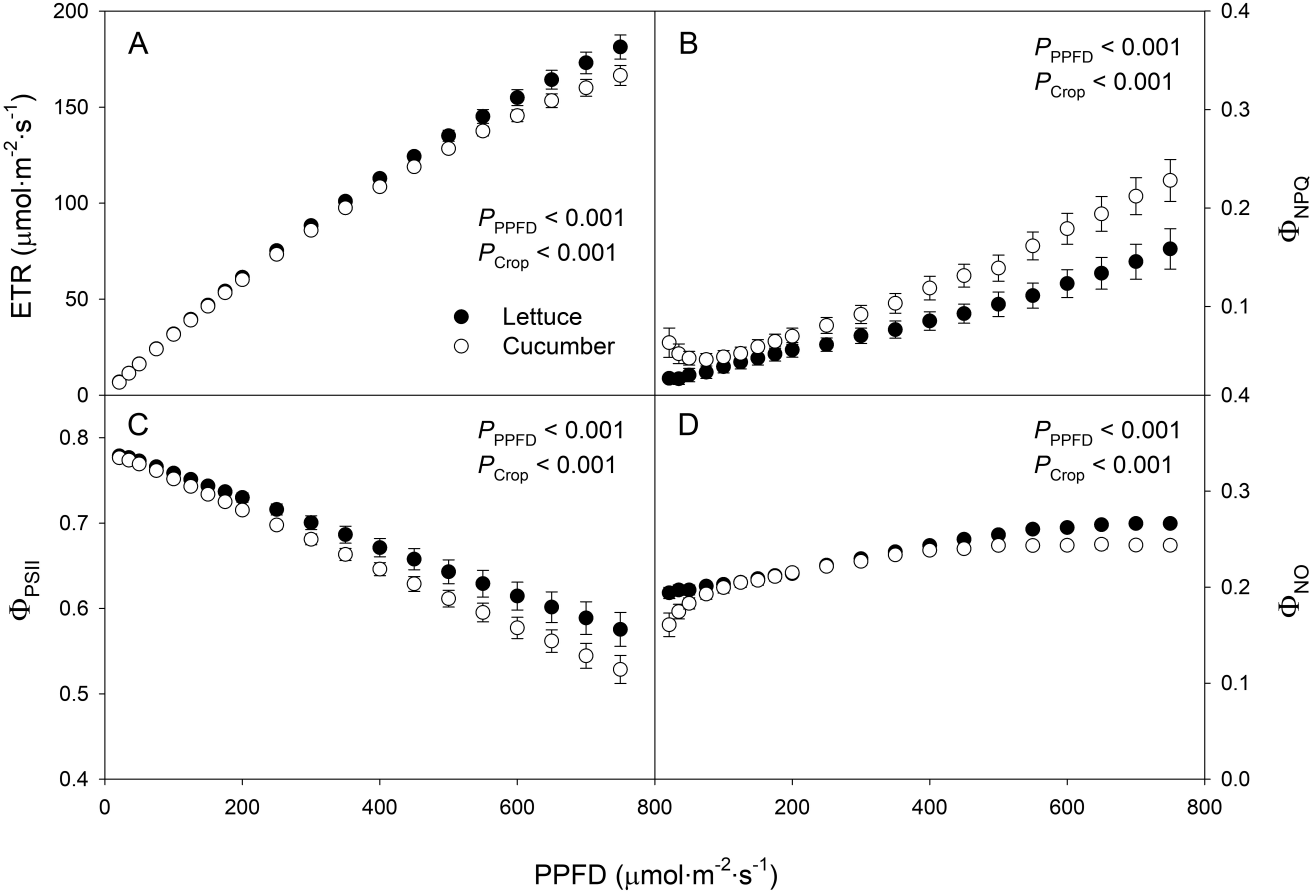
Light response curves for dark‐adapted lettuce (*Lactuca sativa*) ‘Green Towers’ and cucumber (*Cucumis sativus*) ‘Diva’ plants were obtained using pulse‐amplitude modulated (PAM) fluorometers (MINI‐PAM II; Heinz Walz). Electron transport rate (ETR) (A), quantum yield of non‐photochemical quenching (Φ_NPQ_) (B), quantum yield of photosystem II (Φ_PSII_) (C), and quantum yield of non‐regulated energy dissipation (Φ_NO_) (D) were collected across a range of photosynthetic photon flux density (PPFD). PPFD increased in 20 steps from 20 to 750 μmol·m^−2^·s^−1^ (20, 35, 50, 75, 100, 125, 150, 175, 200, 250, 300, 350, 400, 450, 500, 550, 600, 650, 700, and 750 μmol·m^−2^·s^−1^), with each step maintained for 15 minutes for acclimation. Chlorophyll fluorescence parameters were recorded at the end of each step. Error bars indicate standard errors (n = 4). Some error bars are not visible due to their minimal sizes.

Light response curves were obtained across a wide range of light intensities to investigate crop‐specific responses in light utilization efficiency. Lettuce had significantly higher ETR (*p* < 0.001) and Φ_PSII_ (*p* < 0.001) compared to cucumber while having lower Φ_NPQ_ (*p* < 0.001) and slightly higher Φ_NO_ (*p* < 0.001) (Figure 5). This trend was consistent in the light response curves and during the single‐day trials. In the ETR‐based biofeedback trial, cucumber had an average Φ_NPQ_ that was 27% higher than lettuce's, with a 3% lower Φ_PSII_ (Figure 3).

### 3.4 Effect of PPFD, crop type, and recovery time on F_v_/F_m_

**FIGURE 6:**
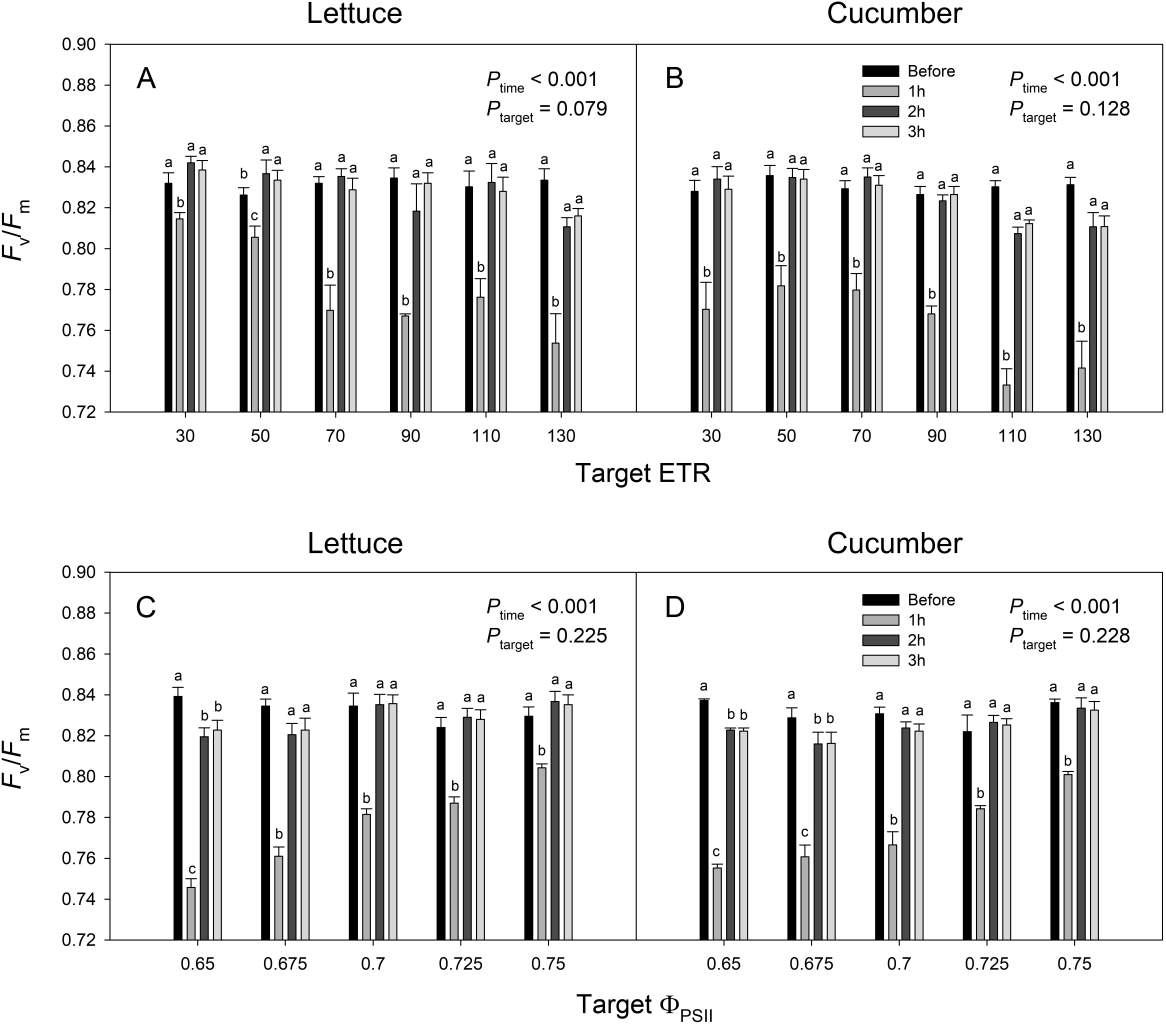
Maximum efficiency of photosystem II (*F*_v_/*F*_m_) in lettuce (*Lactuca sativa*) ‘Green Towers’ and cucumber (*Cucumis sativus*) ‘Diva’ were measured before the photoperiod and 1, 2, and 3 hours after the photoperiod. *F*_v_/*F*_m_ values were compared among the target electron transport rate (ETR) (A and B) and the target quantum yield of photosystem II (Φ_PSII_) (C and D). Each bar represents means ± standard error (n = 4). Mean separation among the dark adaptation periods followed analysis of variance (ANOVA) with Tukey's honestly significant difference (HSD) test.

Lettuce and cucumber plants were initially grown under a 250 μmol·m^−2^·s^−1^ and then transferred to the biofeedback growth chamber. The initial *F*_v_/*F*_m_ was recorded 8 hours after a dark period, assuming all plants were healthy and fully dark‐adapted. The average *F*_v_/*F*_m_ before the light treatments (*F*_v_/*F*_m_ B) was 0.831, with no values falling below 0.8 (Figure 6). Following exposure to varying PPFD levels based on target ETR and Φ_PSII_ treatments, *F*_v_/*F*_m_ was measured for 1 hour (*F*_v_/*F*_m_ 1 h), 2 hours (*F*_v_/*F*_m_ 2 h), and 3 hours (*F*_v_/*F*_m_ 3 h) after the LED lights were turned off. *F*_v_/*F*_m_ 1 h (average 0.773) significantly decreased from the *F*_v_/*F*_m_ B in all treatments for both crops. However, *F*_v_/*F*_m_ 2 h (average 0.827) and *F*_v_/*F*_m_ 3 h (average 0.827) showed significant recovery compared to *F*_v_/*F*_m_ 1 h. In most treatments, *F*_v_/*F*_m_ 2 h and 3 h were not significantly different from *F*_v_/*F*_m_ B, indicating that the maximum photosynthetic efficiency was largely restored within 2 hours. Nevertheless, *F*_v_/*F*_m_ 2 h and 3 h for some low target Φ_PSII_ treatments remained lower than *F*_v_/*F*_m_ B, suggesting slower recovery due to excessive light stress (Figure 6C,D).

**FIGURE 7:**
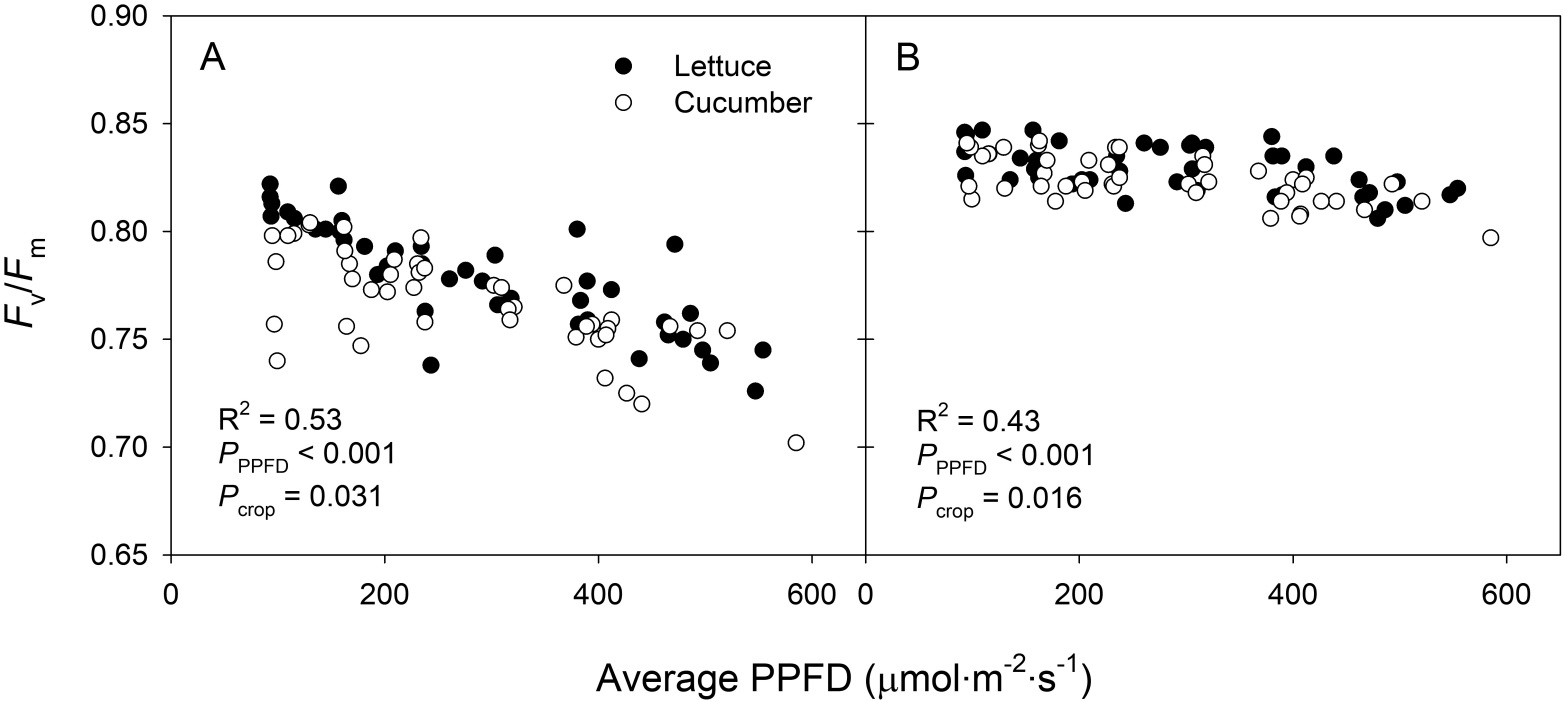
Maximum efficiency of photosystem II (*F*_v_/*F*_m_) in lettuce (*Lactuca sativa*) ‘Green Towers’ and cucumber (*Cucumis sativus*) ‘Diva’ were measured one hour (A) and three hours (B) after the photoperiod. Each *F*_v_/*F*_m_ data point from all target electron transport rate (ETR) and quantum yield of photosystem II (Φ_PSII_) was plotted (n = 4) against the average photosynthetic photon flux density (PPFD) during the day.

The *F*_v_/*F*_m_ 1 h and *F*_v_/*F*_m_ 3 h were plotted against the average PPFD during the 16‐hour light period (Figure 7). *F*_v_/*F*_m_ 1 h showed a substantial decrease with increasing average PPFD (*p* < 0.001), while *F*_v_/*F*_m_ 3 h also declined slightly but significantly with increasing PPFD (*p* < 0.001). Although *F*_v_/*F*_m_ B had no difference between lettuce and cucumber (*p* = 0.46), *F*_v_/*F*_m_ 1 h (*p* = 0.031) and *F*_v_/*F*_m_ 3 h (*p* = 0.016) were higher in lettuce compared to cucumber (Figure 7).

## 4 DISCUSSION

### 4.1 Optimizing saturating light pulses for accurate chlorophyll fluorescence utilization

Applying a saturating light pulse is essential to obtain chlorophyll fluorescence parameters such as Φ_PSII_, *F*_v_/*F*_m_, and ETR. These pulses allow the measurement of maximum fluorescence *F'*_m_ and *F*_m_, which are crucial for calculating these parameters (Maxwell & Johnson, 2000). During a brief and intense saturating flash of light, all PSII reaction centers are temporarily closed, resulting in zero photochemical quenching. The intensity and duration of these pulses must be sufficient to close the majority of PSII reaction centers (Murchie & Lawson, 2013). Most commercial fluorometers provide saturating light pulse intensities between 4,000 and 8,000 μmol·m^−2^·s^−1^. Prolonged saturating light pulses, especially with continuous actinic light, can increase leaf surface temperature, reducing fluorescence yield (GmbH, 1999). We recognize a trade‐off: frequent monitoring of chlorophyll fluorescence parameters is critical for real‐time control of growing conditions, yet accumulated stress from saturating light pulses can lead to photoinhibition, ultimately underestimating actual photosynthetic performance.

To mitigate these effects, researchers may consider reducing the frequency of saturating light pulses. For instance, van Iersel et al. (2016b) monitored *F*_t_ every 5 minutes while measuring *F'*_m_ less frequently at 15‐minute intervals, as *F'*_m_ changes more slowly. In dynamic greenhouse environments, *F*_t_, which does not require a saturating pulse, could be measured more frequently to adjust PPFD in real time, assuming *F'*_m_ remain stable over each 15‐minute interval.

In this study, we tailored the duration and intensity of the saturating light pulse on the MINI‐PAM device settings to reach a maximal fluorescence plateau (Figure 2). The *F'*_m_ measurement interval was set to every 15 minutes during the photoperiod and every 60 minutes at night to reduce photoinhibition. This approach minimized photoinhibition effects on Φ_PSII_ measurements, allowing the biofeedback system to make appropriate PPFD adjustments using the Φ_PSII_‐based logic, which was previously considered less feasible for crop production (van Iersel et al., 2016a).

For long‐term biofeedback system use, accumulated stress from repetitive saturating light pulses can be further mitigated by periodically replacing the measured leaf spot. Monitoring *F*_v_/*F*_m_, a stress indicator, at night can help assess photoinhibition; if *F*_v_/*F*_m_ drops below a threshold (e.g., 0.82), switching to a new, fully expanded leaf can help reduce the stress. However, optimal settings of the saturating light pulse may vary depending on crop type, leaf age, and specific chlorophyll fluorometer device. Therefore, optimizing saturating light pulse properties allows reliable monitoring of photosynthetic performance and effective control of growing light conditions.

### 4.2 The performance of the biofeedback system

The biofeedback system successfully maintained the target Φ_PSII_ and ETR values throughout the experiment period with minimal variation among different plant samples, indicating its ability to maintain specific levels of photosynthetic performance precisely (Figures 3 and 4). In the previous study, the biofeedback system maintained target ETRs of 70, 100, and 125 μmol·m^−2^·s^−1^, adjusting PPFD from 250 to 500 μmol·m^−2^·s^−1^ (van Iersel et al., 2016b). Our study validated that the biofeedback system could also accurately maintain a wider and closely spaced PPFD range from 100 to 500 μmol·m^−2^·s^−1^.

As well as the previous study on the biofeedback system, this study also showed that the ETR and Φ_PSII_‐based biofeedback logics required approximately 30 minutes and 2 hours, respectively, to stabilize the target values (van Iersel et al., 2016a). When dark‐adapted plants are transferred from darkness to light, electron acceptors become occupied, temporarily closing reaction centers and decreasing photochemical efficiency, a phenomenon known as the Kautsky effect (Maxwell & Johnson, 2000). Under continuous light exposure, Φ_PSII_ stabilizes through multiple processes occurring at different time scales. As the light‐induced enzymes in carbon metabolism activate and stomata progressively open, accepted electrons are transported away from PSII for carbon fixation. Additionally, photorespiration, nitrate and sulfate reduction, and the upregulation of NPQ contribute to the slow stabilization of Φ_PSII_, which can take up to 20 minutes, although this varies by species (Elkins & van Iersel, 2020). To prevent excessive up‐ or down‐regulation of PPFD due to the slow stabilizing of Φ_PSII_, predetermined and constant PPFDs need to be provided for the initial 30 minutes without biofeedback control.

The primary advantage of the biofeedback system over conventional lighting strategies is its ability to provide physiologically meaningful amounts of light energy instead of fixed PPFD levels. Previous studies have shown that chlorophyll fluorescence applications have the potential to optimize crop production, particularly in controlling light environments (Ahlman et al., 2017; Schapendonk et al., 2006). Shade screens and supplemental lighting can be managed by monitoring Φ_PSII_, which is influenced by temperature stress, light conditions, and acclimation ability (Baker & Rosenqvist, 2004). The daily photochemical integral was estimated from diurnal chlorophyll fluorescence monitoring data and utilized for controlling supplemental light intensities (Weaver et al., 2019). This strategy achieves not only DLI recommendations but also specific daily crop growth.

The biofeedback system can automatically adjust PPFD based on the real‐time plant physiological responses through serial communication between the fluorometer and the datalogger (van Iersel et al., 2016b). Unlike previous studies focusing solely on monitoring photochemical performance using chlorophyll fluorescence, the biofeedback system uses this information to automatically calculate and adjust appropriate light amount, thereby influencing plant responses again. While conventional lighting strategies often include automatic control of light intensity based on real‐time sunlight levels, they typically do not account for other environmental factors such as temperature, humidity, and acclimation status (van Iersel & Gianino, 2017).

Depending on the objectives and strategies of crop production, the appropriate biofeedback logic can be selected. The ETR‐based biofeedback logic achieves a specific amount of photochemical energy for carbon fixation. When photochemical efficiency decreases due to environmental stresses, PPFD is increased to compensate for the lower efficiency, ensuring a consistent ETR. This guarantees steady growth, making crop growth more predictable. Since ETR increases with PPFD, the ETR‐based logic can adjust PPFD more stably than the Φ_PSII_‐based logic. Conversely, the Φ_PSII_‐based biofeedback might be more intuitive if energy efficiency is the primary concern. The Φ_PSII_‐based logic increases PPFD when plants are well‐acclimated to optimal conditions and decreases PPFD under stressful conditions. Providing supplemental light when it will be most efficiently used for photosynthesis can reduce electricity costs by controlling light from the plant's physiological perspective (Weaver et al., 2019).

### 4.3 Crop‐specific acclimation responses to high light intensities

Lettuce and cucumber plants exhibited distinct acclimation responses to high light intensities. All plants were initially grown under moderate light conditions of 250 μmol·m^−2^·s^−1^, and they were subsequently exposed to a range of PPFD levels during the experiment. Lettuce plants subjected to the highest target ETR initially showed very low Φ_PSII_ but gradually recovered over the 16‐h photoperiod, with a slight decrease in Φ_NPQ_ over the course of a day (Figure 3E). High‐light acclimation involves both rapid and long‐term mechanisms. Upon a sudden shift from low to high light intensities, plants respond within seconds to minutes by increasing thermal energy dissipation through NPQ, reorganizing the photosynthetic electron transport chain, and altering redox states and reactive oxygen species (ROS) levels. These photoprotective responses mitigate excessive photoinhibition at the expense of reduced quantum efficiency for photochemistry (Dietz, 2015).

Under sustained high‐light conditions, longer‐term acclimation processes enable the recovery of photosynthetic efficiency on a scale of hours. This acclimation includes optimizing the photosynthetic machinery by reducing PSII antenna size and enhancing D1 protein synthesis to support the repair cycle. Additionally, genes associated with photosynthesis are upregulated, while signaling pathways involving plant hormones, such as abscisic acid (ABA), coordinate responses to prolonged light stress (Dietz, 2015; García‐Plazaola et al., 2002; Wientjes et al., 2013).

Cucumbers under target ETR levels of 110 and 130 μmol·m^−2^·s^−1^ showed a gradual decrease in Φ_PSII_ and a corresponding increase in Φ_NPQ_ over time (Figure 3F,H). Light response curves indicated that cucumbers consistently had lower Φ_PSII_, higher Φ_NPQ_, and similar Φ_NO_ compared to lettuce under the same PPFD. This implies that cucumber dissipated more light energy as heat through regulated mechanisms, which reduced photochemical efficiency and electron transport for carbon assimilation (Figure 5).

Previous studies have also shown varying photosynthetic efficiencies and light adaptation abilities among different crops (Jayalath & van Iersel, 2021; Zhen & van Iersel, 2017). Sun‐demanding species generally exhibit greater photoprotective capacities, with higher NPQ, greater xanthophyll cycle pigment amount, and larger plastoquinone pool size compared to shade‐adapted crops (Kromdijk & Walter, 2023; Shuang et al., 2022). Cucumber might have had a more active xanthophyll cycle, leading to sustained and increased NPQ alongside relatively lower Φ_PSII_. This robust NPQ response in cucumbers likely contributes to their constantly lower *F*_v_/*F*_m_ compared to lettuce (Figure 7).

Non‐regulated energy dissipation, represented by Φ_NO_, decreased over time in both lettuce and cucumber (Figure 3I,J). Φ_NO_, indicative of passive energy dissipation as heat and fluorescence, can contribute to ROS production and potential damage to PSII. As such, Φ_NO_ serves as a stress indicator, reflecting insufficient photoprotective capacity (Bürling et al., 2010). During acclimation to high light, both crops shifted their energy utilization towards either photochemistry or regulated heat dissipation, thereby reducing dependence on non‐regulated dissipation mechanisms.

The biofeedback system effectively tracked real‐time changes in photosynthetic efficiency and crop‐specific responses and adjusted LED light intensities accordingly. For lettuce at target ETR 130 μmol·m^−2^·s^−1^, the required LED light intensity decreased as Φ_PSII_ increased, indicating potential savings in electricity costs as the plants acclimated to high light conditions while maintaining consistent photochemical activity (Figure 3C,E). On the other hand, the Φ_PSII_‐based biofeedback system required higher PPFD for lettuce compared to cucumber under the same target Φ_PSII_. This suggests crops with greater photosynthetic efficiency can be provided with higher light intensity while having the same Φ_PSII_ level (Figure 4C,D). Zhen and van Iersel (2017) emphasized the importance of considering light acclimation and crop‐specific responses in supplemental light control strategies to enhance light use efficiency. There were significant differences in ETR among sun‐ and shade‐acclimated plants and between crop species, which may impact differences in carbon assimilation and crop yield. Another study found that gradually increasing PPFD during cultivation optimized light use efficiency and increased final shoot biomass compared to strategies constantly decreasing PPFD (Jin et al., 2023). Therefore, tailoring the target ETR and Φ_PSII_ based on acclimation status and crop type can enhance the effectiveness of the biofeedback system throughout the cultivation period.

### 4.4 Monitoring F_v_/F_m_ for investigating environmental stress and photoinhibition

*F*_v_/*F*_m_ represents the theoretical and empirical maximum quantum efficiency of PSII when all reaction centers are open. Healthy, non‐stressed leaves typically have an *F*_v_/*F*_m_ value around 0.83, which is highly consistent across plant species. Various abiotic stressors, such as drought, extreme temperatures, and high light intensity, can induce sustained quenching or damage in PSII, leading to a decrease in *F*_v_/*F*_m_ (Fracheboud & Leipner, 2003). Thus, *F*_v_/*F*_m_ is commonly used as a stress indicator following adequate dark adaptation (Murchie & Lawson, 2013). Under excessive light conditions, the absorption of light energy can surpass the capacity for photochemical utilization, leading to overexcitation of PSII. This condition may produce ROS and cause damage to PSII components such as D1 protein. A decline in *F*_v_/*F*_m_ may indicate photoinhibition, which may be reversible with recovery or permanent if the stress is severe or prolonged (Krause, 1988). Chen et al. (2022a) reported that *F*_v_/*F*_m_ in lettuce declined as PPFD increased, supposedly because the PSII repair mechanism requires more time to recover from severe photodamage (Takahashi & Badger, 2011). Likewise, in this study, both lettuce and cucumber plants had lower *F*_v_/*F*_m_ 1 h under higher target ETR and lower target Φ_PSII_, indicating that accumulated light stress during the day resulted in photoinhibitory conditions (Figure 7).

However, a decline in *F*_v_/*F*_m_ does not necessarily indicate photoinhibition. Instead, it can reflect tentative and reversible NPQ mechanisms, particularly thermal deactivation in the pH‐ and energy‐dependent quenching (qE) component. qE is responsible for safely dissipating excess light energy as heat through the proton gradient and the xanthophyll cycle (Muller et al., 2001). Given that most NPQ consists of the qE component, which dissipates within seconds to minutes, 20–30 minutes of dark adaptation is generally sufficient to assess photoinhibition from environmental stresses. However, a longer dark adaptation may be necessary in severe and prolonged suboptimal conditions. Photoinhibition can be reversible through a restoration process that takes place up to hours, related to the photo‐inhibitory quenching (qI) component (Krause, 1988). If the pre‐dawn *F*_v_/*F*_m_ after several hours of dark adaptation is still lower than approximately 0.83, the plant has likely experienced substantial photoinhibition or permanent damage to PSII (Murchie & Lawson, 2013).

The dark adaptation period for *F*_v_/*F*_m_ measurements needs to be carefully determined based on the experimental environment and objectives (Murchie & Lawson, 2013). The duration required for full dark adaptation to resolve reversible photoinhibition may vary depending on the extent of PSII damage (Chen et al., 2022a). Murchie and Lawson (2013) recommended frequent measurements of *F*_v_/*F*_m_ until a steady value is achieved to ensure accurate assessments of plant adaptation.

In this study, as the average PPFD increased, *F*_v_/*F*_m_ decreased drastically to as low as ~0.72 after one hour of dark adaptation. However, the plants recovered from the high light stress, with *F*_v_/*F*_m_ values returning to above 0.8 after three hours of dark adaptation (Figure 7). A short dark adaptation period of 20–30 minutes was sufficient to relax the majority of sustained NPQ and recover from the down‐regulation of PSII. However, a few more hours of dark adaptation might be necessary to determine if plants have fully adapted to the conditions or if excessive damage is impeding photosynthesis and proper growth. In this context, the biofeedback system is valuable for continuous real‐time monitoring of *F*_v_/*F*_m_, enabling the determination of when plants are fully dark‐adapted and assessment of the extent of photoinhibition.

### 4.5 Limitations, future works, and applicability of the Biofeedback system

Chlorophyll fluorescence measurement is a useful tool for estimating photosynthetic performance due to its simplicity, non‐invasiveness, and time efficiency. The linear electron transport rate in PSII, measured by pulse amplitude modulation (PAM) fluorometry, is strongly correlated with the CO_2_ assimilation rate. However, discrepancies can arise under stress conditions. For example, high temperatures or drought stress can allocate electron transport to alternative sinks, such as photorespiration, instead of carbon fixation, potentially causing Φ_PSII_ and ETR measurements to overestimate photosynthetic performance (Maxwell & Johnson, 2000; Vongcharoen et al., 2019). This limitation can lead to inaccurate adjustments of LED light intensities by the biofeedback system. Another drawback is the high cost of the instruments available to measure chlorophyll fluorescence, which makes large‐scale application of this technology challenging.

The low frequency of PPFD adjustments can be another limitation of the biofeedback system, while some conventional strategies can adjust LED light intensities every second based on ambient sunlight levels. The challenge of frequent Φ_PSII_ measurements arises because the continuous application of saturating light pulses can stress the measured leaf spot. While frequent adjustments are less critical in stable indoor environments like vertical farms, they are necessary in greenhouses with varying conditions to achieve target chlorophyll fluorescence parameters. To address these limitations, passive and indirect techniques such as remote sensing, chlorophyll fluorescence imaging, reflectance measurement, and sunlight‐induced chlorophyll fluorescence, which do not induce photoinhibition, offer promising alternatives for controlling light intensities on a larger scale (Murchie and Lawson, 2013). These methods could be integrated into the biofeedback system to facilitate more frequent PPFD adjustments without compromising the photochemical ability of plants (Durmus, 2020; Peñuelas et al., 1995; van Iersel, 2017).

The biofeedback system was tested for 16 hours in this study to investigate how the system maintains a wide range of target Φ_PSII_ and ETR while adjusting PPFD in a short period. For future work, long‐term trials should be considered to examine longer acclimation responses and to demonstrate the system's applicability throughout an entire crop growth cycle. Unlike conventional lighting strategies, the biofeedback system considers both light requirements and physiological responses to various environmental factors. It will be important to evaluate how the biofeedback system adjusts supplemental light under different temperatures or humidity, and consequently VPD. Chen et al. (2022b) observed higher Φ_PSII_ and ETR in the morning compared to the afternoon on hot summer days, indicating diurnal changes in photosynthetic efficiency. The ETR‐based biofeedback system will automatically increase supplemental light intensity in stressful conditions to compensate for reduced photosynthetic capacity, whereas the Φ_PSII_‐based system will decrease light intensity to apply supplemental light only when plants can use it efficiently. Therefore, the biofeedback system is particularly beneficial under greenhouse conditions with dynamic and fluctuating environmental factors, not limited to indoor facilities (van Iersel et al., 2016b).

## 5 CONCLUSIONS

This study validated the technical feasibility and applicability of the biofeedback system, establishing it as a reliable LED light control strategy. It demonstrated the effectiveness of the biofeedback system in maintaining target Φ_PSII_ and ETR levels by dynamically adjusting LED light intensities in real‐time. The ETR‐based biofeedback logic ensures consistent photosynthetic activity and growth, while the Φ_PSII_‐based logic optimizes light use efficiency, potentially reducing electricity costs. Distinct high light acclimation responses were observed between lettuce and cucumber, with the biofeedback system adjusting LED intensities according to crop‐specific responses and acclimation status over time, even under constant environmental conditions. This innovative approach has the capacity to outperform conventional light control strategies by providing physiologically meaningful light quantities rather than fixed amounts. This study also offers a strong foundation for further research on the biofeedback system aimed at greenhouse applications and larger‐scale CEA systems.

## AUTHOR CONTRIBUTIONS

Conceptualization and experimental design, S.N. and M.W.v.I.; data acquisition, S.N.; data analysis, S.N.; visualization, S.N. and R.S.F.; draft writing, S.N. and R.S.F.; supervision, R.S.F. and M.W.v.I.; funding acquisition and project administration, R.S.F. and M.W.v.I.; critical revision of the manuscript, R.S.F.

## FUNDING INFORMATION

This research was funded by the USDA‐NIFA‐SCRI, award number 2018–51181‐28365, project ‘LAMP: Lighting Approaches to Maximize Profits’, the Department of Horticulture, the College of Agricultural and Environmental Sciences, and the Office of the Senior Vice President for Academic Affairs and Provost.

## Data Availability

The data that support the findings of this study are available from the corresponding author upon reasonable request.
